# Ideal free distribution of *Daphnia* under predation risk—model predictions and experimental verification

**DOI:** 10.1093/plankt/fby024

**Published:** 2018-07-03

**Authors:** Piotr Maszczyk, Ewa Babkiewicz, Marta Czarnocka-Cieciura, Z Maciej Gliwicz, Janusz Uchmański, Paulina Urban

**Affiliations:** 1 Department of Hydrobiology, Faculty of Biology, University of Warsaw at Biology and Chemistry Research Centre, Żwirki i Wigury 101, Warsaw, Poland; 2 Faculty of Christian Philosophy, Institute of Ecology and Bioethics, Cardinal Stefan Wyszynski University, Wóycickiego 1/3, Warsaw, Poland; 3 Laboratory of Functional and Structural Genomics, Center of New Technologies, University of Warsaw, Banacha 2c, Warsaw, Poland; 4 College of Inter-Faculty Individual Studies in Mathematics and Natural Sciences, University of Warsaw, Banacha 2c, Warsaw, Poland

**Keywords:** *Daphnia*, fitness, functional response, ideal free distribution, predation risk

## Abstract

The vertical distribution of planktonic animals, such as *Daphnia*, in overlapping gradients of food concentration and risk of visual predation should depend on *Daphnia* population density and should be the result of the group effect of optimizing decisions taken by each individual (juvenile or adult), trading-off a high growth rate to low mortality risk. We tested this hypothesis by comparing the theoretical distributions from simulations based on an experimentally parameterized, optimizing individual-based model (consistent with the assumptions of the concept of the interference ideal free distribution with costs) with distributions observed in laboratory experiments. The simulations were generated for two scenarios, where the shape of the functional response of fish is consistent with either type II or III. The results confirmed the hypothesis. The greatest similarity of the distributions obtained in the experiments and simulations was found for the simulations based on the scenario assuming the type III rather than type II for both age classes of *Daphnia*. This was consistent with the results of the experiments for the model parameterization, which revealed the type III functional response of fish. Therefore, the results suggest that aggregating may be maladaptive as an anti-vertebrate-predation defense in the case of zooplankton.

## Introduction

The vertical distribution of planktonic animals in the water column of a lake or ocean is generally uneven (Lampert, 2011). This unevenness may be partially influenced by random physical factors, such as wind-driven water currents (Pinel-Alloul, 1995; Folt and Burns, 1999). However, it is most often explained as the result of individual decisions, where each animal in the population attempts to optimize its vertical location in overlapping gradients of biotic (e.g. food concentration and predation risk) and abiotic conditions (temperature, UV radiation and oxygen concentration) to achieve the best chances of survival and net energy gain (Zaret and Suffern, 1976; Williamson *et al.*, 1996; De Meester *et al.*, 1999).

The literature provides numerous examples of studies indicating that population density is an important variable affecting the distribution of a variety of vertebrates (Lima *et al.*, 1985; Heithaus and Dill, 2002; Skalski and Gilliam, 2002) and invertebrates (Jakobsen and Johnsen, 1988; Gliwicz *et al.*, 2006). This is because both net energy gain (Fretwell and Lucas, 1970) and predation risk (Holling, 1959) may depend on population density. In the case of zooplankton, distribution was revealed to be greater even at high population densities, when individuals were distributed in the food gradient alone (Larsson, 1997) or in the overlapping gradients of food and temperature (Lampert, 2005). This suggests that crowding has a rather negative effect in terms of resource acquisition. The literature on the effect of population density on the distribution of zooplankton in predation risk gradients is less consistent. Some studies revealed that planktonic animals, such as the *Daphnia* cladoceran order, create more tight aggregations in the presence of predation risk to visually oriented planktivorous fish (Jakobsen and Johnsen, 1988; Pijanowska, 1994), and that fish are more confused in prey aggregations (Milinski and Heller, 1978). This would suggest that a high population density could be treated as an anti-predation defense. Therefore, it could be expected that at higher population densities, *Daphnia* select more illuminated, but also more profitable, in terms of resource acquisition, depths closer to the surface due to reduced mortality risk. On the other hand, some studies (Gliwicz *et al.*, 2006, 2012) suggest that aggregating may be maladaptive as an anti-predation defense, as fish increase (behaviorally and numerically) their foraging activity in patches of prey (McNamara and Houston, 1985). Therefore, it could be expected that at higher population densities, *Daphnia* select deeper strata to reduce the risk of mortality. Moreover, literature does not provide any example, in which the effect of population density was tested in the overlapping gradients of food concentration and predation risk, when individuals trade-off the maximization of density dependent net energy gain and the minimization of density-dependent predation risk in their quest to find the most profitable location.

A good starting point (null model) to examine the density-dependent distribution of animals in a population is the concept of ideal free distribution (IFD, Fretwell and Lucas, 1970). The classical version predicts the optimal distribution of individuals along a gradient of the input of resources, when subsequent portions of resources arrive in each patch continuously and are consumed immediately by the competitors waiting there. One of its modified versions—Interference IFD (Tregenza, 1995) assumes a stable (independent of population density) amount of resources, rather than a constant input. This simple theory is based on several controversial assumptions: (i) each individual can ideally assess food profitability at each location and choose the one that offers the highest possible food intake, (ii) the individual is assumed to be free to move between sites without any movement costs, (iii) its competitive ability is the same as that of all others and (iv) energy gain is density dependent. When these assumptions are fulfilled in a classical continuous input model, the distribution of individuals at equilibrium should reflect the input of resources. In the case of IIFD, the distribution of individuals in the gradient of resources will depend on the strength of the interference between individuals, included in the model as an interference parameter, (Sutherland, 1983). When the value of this parameter is near zero (interactions between individuals are negligible), all individuals should choose the location with the highest concentration of resources. When the value of this parameter is equal to 1, the distribution of individuals should reflect the distribution of resources, and when the value is more than 1, individual distribution should be more uniform than the distribution of resources. In each of the two scenarios, the net energy gain (a measure of fitness in the absence of predation) of each individual in every patch is the same and maximal, which means that the distribution is evolutionarily stable.

In the presence of fish predation, *Daphnia* population density is usually below the carrying capacity level. Thus, the net energy gain of *Daphnia* within a gradient of algal food alone should depend on the strength of the interactions between individuals and the concentration, rather than the input of algae. This is why the distribution would be predicted by the IIFD model rather that by continuous input models. Although in an early study, Larsson (1997) tested the IFD of *Daphnia* in a horizontally oriented gradient of algal food, this work incorrectly predicted that their distribution should reflect the distribution of the food (continuous input model), even though the resource gradient was stable (interference model scenario). *Daphnia* distribution could also mimic the distribution of resources if interference is weak, but individuals have to move to test the ambient conditions because of perceptual constraints (Abrahams, 1986; Dall et al., 2005; Sih and Del Guidice, 2012). Using inappropriate model predictions does not allow the testing of whether the distribution at the equilibrium state is evolutionarily stable (i.e. all individuals achieve the same maximal fitness). Nevertheless, this study provided evidence that the distribution in an algal gradient is density dependent by its indication that the distribution of *Daphnia* at a higher density is more uniform with the same food distribution (Larsson, 1997). Also, the study by Lampert *et al.* (2003) incorrectly assumes that animal distribution should mimic the fitness gradient (calculated without taking into account density effects), even though the food resource gradient was stable (interference model scenario), and temperature, also incorporated in the model (model of IFD with costs; Tyler and Gilliam, 1995) was treated as a non-depletable resource. Although the distribution in experiments with the two gradients mimicked the model predictions, it is likely that other factors (e.g. perceptual constraints) rather than competition between individuals, caused some *Daphnia* not to choose a location where fitness would be the highest if no competition occurred. Thus, the results of these studies do not verify the hypothesis of density-dependent optimal *Daphnia* distribution. However, one study (Lampert, 2005) provided evidence that *Daphnia* density affects its distribution in food and temperature gradients. At a higher density, the distribution became more uniform. However, this study did not attempt to verify how the gradient of predation risk modifies the distribution resulting either from the gradients of temperature and food or the density-dependent food gradient alone.

Predation risk can be also treated as a form of cost in the modified version of IFD. While individual growth rate is usually sufficient to apply as a proxy of fitness along the gradients of food abundance and temperature (Lampert *et al.*, 2003; Kessler and Lampert, 2004), the growth rate to mortality risk ratio seems to be more appropriate when seeking the fitness of an individual facing the gradients of both food abundance and mortality risk at the same time (e.g. Hugie and Dill, 1994; Giske *et al.*, 1997), as is clear from the mortality risk to growth rate minimizing rule of Werner and Gilliam (Werner and Gilliam, 1984).

As the mortality risk may depend on population density, one should expect that the functional response of a predator (the relationship of the consumption rate of the prey and the density of their population, Holling, 1959) should influence the distribution of *Daphnia*. The functional response of fish could be type II or III (Townsend and Risebrow, 1982; Winkler and Orellana, 1992; Kempf *et al.*, 2008). In the case of type II, the highest mortality risk is at the lowest prey density, so one should expect the greatest modification of the distribution according to IFD predictions in the food gradient alone at the lowest prey density. For type III, the highest mortality risk is at moderate prey density, so individuals have low and high-density refuge, thus one should expect the greatest modification of the distribution at moderate densities.

The IFD model could also be extended to cover a different growth rate and mortality risk for individuals in different ontogenetic stages and thus with different body sizes. Body size is crucial because the optimal depth distribution is obviously not the same for all ontogenetic stages in any combination of the two gradients (Stich and Lampert, 1981; Hays *et al.*, 2001; Kessler, 2004; Kessler and Lampert, 2004). It is also possible to construct a model of IFD with a relaxed assumption that *Daphnia* are omniscient, so that they are perfectly able to detect the conditions at every depth.

The aim of the study was to test the concept of IIFD with costs in two age classes of *Daphnia* (juveniles or adults) in overlapping gradients of food concentration and risk of predation by planktivorous fish, both the highest in subsurface layers. This hypothesis was tested by comparing the theoretical distribution from simulations based on an experimentally parameterized optimizing individual-based model (IBM) with the distributions observed in laboratory experiments performed in a small-scale experimental setup consisting of twin vertical columns.

## Method

### The approach

The hypothesis was verified for adult and juvenile *Daphnia longispina* in a five-step approach: (Step 1) establishing the conditions in the experiments (gradients, *Daphnia* clones, population density range, and age classes); (Step 2) constructing a simulation, optimizing individual-based interference IFD model with costs (IIFDC) taking into account the type II or III functional responses of fish; (Step 3) performing four types of experiments to determine the parameters for the model, including experiments to assess: (i) the relationship of individual *Daphnia* growth rate and the concentration of algal food, the population density of *Daphnia*, the age of an individual, and the light intensity, (ii) the relationship of the reaction distance (RD) of planktivorous fish (with respect to each of the two *Daphnia* age classes) and the intensity and spectral composition of the light, (iii) the relationship of *per capita* mortality risk from fish and *Daphnia* population density (juveniles or adults) and (iv) the slowdown of the *Daphnia* growth rate (juvenile and adult) in the food gradient resulting from imperfect knowledge about the resources available at each location; (Step 4) performing experiments to verify the model predictions in the apparatus comprised of small-scale twin vertical water columns (Maszczyk, 2016), in gradients of algal food and information on predation risk (gradients of light intensity and spectrum and the presence of chemical signals on fish threat—kairomones and alarm substances); (Step 5) generating simulations of virtual distributions for both age classes of *Daphnia* in both versions of the model assumptions and confronting these distributions with those obtained in the experiments under the same experimental conditions.

### Establishing the conditions in the experiments (Step 1)

Experimental conditions were established based on measurements and samples taken from the epilimnion of a typical dimictic eutrophic lake, Lake Roś (Great Mazurian Lakes, Poland), on a cloudy day in September 2012. In each experiment, the temperature was maintained at 21°C and the oxygen concentration was close to the saturation level (9 mg O_2_ × L^−1^), which mimics the conditions in the top 10 meters of the water column in the lake.

In the majority of the experiments, medium-sized *Daphnia longispina* (clone L004 isolated from the metalimnion of Lake Roś in September 2012) was used, as it is a common and typical planktonic animal in the pelagial of European lakes. The only exception were the experiments conducted to determine the relationship of *per capita* mortality risk from planktivorous fish and zooplankton population density (Step 3c). In these experiments, 2-day-old *Artemia franciscana* (Sanders®, Great Salt Lake, Utah, USA) was used as a substitute for the *Daphnia*. The body mass of two-day-old *Artemia* is similar to the body mass of juvenile *Daphnia* (4.9 and 5.3 μg of dry weight, respectively). In each experiment, *Daphnia* (or *Artemia* as its substitute) were added to the experimental systems at densities reflecting their natural densities in the lake (between 1 and 60 ind. × L^−1^). Experiments were performed with two age classes of *Daphnia*, either 2.5- or 5.5-day-old individuals (juveniles or adults, respectively). Older individuals were at the stage of just before depositing the first clutch of eggs in the brood pouch.

In the experiments with fish, we used tap water stored for 24 h before each experiment. In the remaining experiments, media were prepared using conditioned water from the eutrophic Lake Góra (Warsaw, Poland), filtered through 0.2 μm Sartorius membrane filters. The water was stored and aerated for at least 14 days before each experiment to remove possible traces of information on conspecifics or predators (kairomones, Loose *et al.*, 1993). We used *Acutodesmus obliguus* from a long-established laboratory culture as a high-quality algal food for the *Daphnia*. Before the experiments, the *Daphnia* were maintained in glass containers with 2.0-μm filtered lake water and fed daily with *Acutodesmus* at a concentration above the incipient food level (0.8 mg C_org._ × L^−1^). Before each experiment, lake water was transferred to 60-L aerated containers and kept at a constant temperature of 21 ± 0.1°C for 24 hour. Juvenile (6–8 cm) common rudd (*Scardinius erythrophthalmus*) were used as a typical European cyprinid fish, either in experiments with chemical signals on fish threat (kairomones) or in experiments with fish. To produce media with kairomones, three fish were introduced into containers (1 fish per 20 L). Fish had been fed once a day with a standard amount of frozen *Chironomidae* larvae and *Daphnia*. Before the media were prepared, water from the containers was filtered through 0.2-μm Sartorius membrane filters.

### The model (Step 2)

The IBM (Uchmański and Grimm, 1996; Grimm and Railsback, 2005) optimizing the behavior of individuals was constructed in the NetLogo programming language (Wilensky, 1999). The justification for using IBM is provided in Appendix 1. The model consisted of virtual individuals moving in a spatially one-dimensional environment looking for the conditions maximizing their fitness function (for the details of the model, see Appendix 1). The model was discrete in space and time and demography was not included in the model (i.e. the age and number of virtual individuals remained constant). The environment consisted of 10 sectors reflecting the construction of the experimental apparatus to study the vertical distribution of zooplankton (Maszczyk, 2016). Sectors were stacked one onto another so that each of them was connected to two others, except for the top and bottom ones, which were connected only to one neighboring sector. In each time step, each individual chosen in random order from the whole number of individuals present in simulation performed one of the two movement options that offered greater fitness—either it stayed in the current sector when no one from adjacent sectors offered greater fitness or it moved to one of the adjacent sectors and stayed in this one in which it had greater fitness. After such calculations, the position of the individual along environmental gradient was changed inside the time step, so the next individual chosen for calculations faced a little different situation than it had before. Each time step consisted of such calculations for all individuals. As a measure of fitness (Eq. (1) in Appendix 1), we used the ratio of individual growth rate to *per capita* mortality rate (Werner and Gilliam, 1984). The justification for using this measurement as a proxy of fitness is provided in Appendix 1. All individuals in each sector were equal (i.e. their fitness depends in the same way on environmental conditions). The end result of the model was the number of individuals in each sector.

The model assumed that each virtual individual has perfect knowledge on the current location, but may wrongfully estimate conditions in the adjacent sectors. The probability of making errors in assessing the growth rate (different for juveniles and adults), and therefore the depth selected by *Daphnia*, was introduced to the model using the threshold difference of food concentration that can be detected by an individual (Appendix 1, Eq. (10–11)). This was estimated from the results of the experiments performed in Step 3d (see below and Appendix 2). The probability of making errors in assessing mortality risk was introduced to the model using the threshold difference of light intensity that can be detected by an individual (Appendix 1, Eq. (12–14)) and was estimated based on data from the literature (Ringelberg *et al.*, 1967).

The relationship between algal food concentration and growth rate was expressed by the asymptote function (Ivlev, 1955), and the relationship between population density and the growth rate for both age classes of *Daphnia* was expressed by the linear function. These relationships were included in one formula (Appendix 1, Eq. (2)). Moreover, the formula also included the relationship between the information on predation threat and growth rate, as well as pairwise interaction between the effect of the information on predation threat and age class on the growth rate. The parameters of this formula were estimated based on “the growth rate experiments” (Step 3a, see below and Appendix 2).

*Per capita* mortality rate was calculated as the sum of mortality caused by fish and other sources (i.e. background mortality, Appendix 1, Eq. (3)). It was assumed that background mortality was constant and did not depend on light intensity, population density or age class. Its value was estimated as 1 × L^−1^, where L is *Daphnia* longevity in 20°C in an environment without fish, which was assumed to be 50 days (Cowgill *et al.*, 1985; Pennak, 1989). Fish-caused mortality was related to the fish’s consumption rate (Appendix 1, Eq. (4)) and calculated using either the type II or III functional response (Appendix 1, Eq. (5)). The type II functional response was based on Holling’s original disc equation (Holling, 1959) and type III was based on the modified disc equation developed by Gliwicz and Wrzosek (2008). It was assumed that the rate of consumption by fish depended on light intensity and its spectral composition, *Daphnia* age class and its density in a particular sector (Appendix 1, Eq. (5–9)). The parameters of the type II function were estimated based on the experiments performed in Steps 3b and 3c (see below and Appendix 2). There were two versions of the model assumptions, considering that the functional response was either type II or type III. Both of the versions were used to simulate the distribution of each single experiment conducted to verify the model predictions.

### Experiments to determine the parameters for the model (Step 3)

#### The growth rate experiments (Step 3a)

The relationship of individual growth rate and the concentration of algal food, the population density of *Daphnia*, the age of the individual and the light intensity were determined based on the results of experiments conducted in a standard flow-through system comprised of 250-ml glass chambers (Stich and Lampert, 1984). A detailed description of the experimental design is provided in Appendix 2.

#### The experiments for assessing the relationship of RD and the intensity and spectral composition of the light (Step 3b)

The relationship of the RD of planktivorous fish (with respect to each of the two age classes) and the intensity and spectral composition of the light was determined based on the results of experiments conducted in the experimental system, which allowed us to precisely observe and register fish foraging behavior at a constant prey density (Bartosiewicz and Gliwicz, 2011). A detailed description of the experimental design is provided in Appendix 2.

#### The experiments for assessing the relationship of *per capita* mortality risk from fish and *Daphnia* population density (Step 3c)

The relationship of *per capita* mortality risk from planktivorous fish and zooplankton population density was determined based on the results obtained from the experiments in the experimental system. This system consisted of a set of 10 tanks of 200-L capacity, interconnected in a loop to allow the free movement of fish, located in an indoor lab (Gliwicz and Maszczyk, 2016). In this experiment, naupli of *Artemia franciscana* were used as a proxy of *Daphnia*—*Artemia* were easier to obtain in large quantities. A detailed description of the experimental design is provided in Appendix 2.

#### The experiments for assessing the slowdown of *Daphnia* growth rate in the food gradient resulting from imperfect knowledge (Step 3d)

The reduction in the growth rate of *Daphnia* resulting from residing in a suboptimal location due to perceptual constraints was determined based on the results of experiments conducted between September and October 2015 in the same apparatus as the main experiment (Step 4). We compared the growth rate of *Daphnia* between two different algal distributions: one column with a maximum food concentration at the top and the other column with the same maximum food concentration homogeneously distributed. Homogenous food conditions simulated the scenario in which *Daphnia* are omniscient in assessing growth rate.

### Experiments aimed at verifying the model predictions (Step 4)

To answer the main hypothesis, experiments were carried out in the twin vertical water columns to study zooplankton distribution (Maszczyk, 2016). Experiments were performed for two age/size classes in four treatments being the combination of the presence or absence of an algal gradient and a gradient of light intensity and spectrum in the presence of fish kairomones and alarming signals. Two experiments were carried out simultaneously. Each experiment was conducted either in the left or right column and lasted 6 hours. *Daphnia* remained without any gradients during the first 2 hours for acclimation, during the next 2 hours in gradients that were gradually established, and finally in stable conditions over the last 2 hours representing one of the four treatments. In total, 74 experiments (8–11 for each treatment and each age class) were conducted at different *Daphnia* densities (between 0.166 and 62 ind. × L^−1^). *Daphnia* distribution was determined as the mean from two counts performed during the last 2 h of the experiments.

Before each experiment, *Daphnia* were maintained in glass containers with 2.0-μm filtered lake water and fed daily with *Acutodesmus* algae at a concentration above the incipient food level (0.8 mg C_org._ × L^−1^). Each experiment started by placing *Daphnia* in the fifth sector from the top of each column. At the end of each experiment with the algal gradient, water samples were taken from each depth of both columns to assess the algal concentration using the fluorometer. *Daphnia* observations were performed using a night vision device coupled with an infrared diode matrix placed between the columns.

### Simulations of virtual Daphnia distributions and the confrontation of the distribution from the experiments and simulations (Step 5)

For each single experiment conducted to verify the model predictions, two simulations of virtual *Daphnia* distributions (each repeated 100 times) were generated, representing two versions of the model assumptions, taking into account the type II or III functional response of planktivorous fish. At the beginning of each simulation, between 1 and 360 individuals were placed in the central sector. All individuals in a single simulation were identical and represented either 2.5- or 5.5-day-old *Daphnia*. In each simulation, *Daphnia* age class, its population density and the environmental conditions (the presence or absence of algal and predation risk gradients) were set to represent one experiment. Each simulation lasted 200 time steps, but only the last 100 was taken into account to skip the phase of attaining the equilibrium. The final distribution of virtual *Daphnia*, used in the comparison with experimental results, was calculated as a mean of 100 last time steps of 100 repetitions of the same simulation. It was easier to reconstruct the experimental conditions in the simulation rather than *vice versa*. Yet the model was still *a priori*, because the results of the main experiment were not used to design or parameterize the model.

### Data analysis

The parameterization of the equations in the model and the statistics used to assess the effect of all variables and the interactions of those equations are described in Appendix 3.

To assess the difference of the distribution of *Daphnia* between 12 different comparisons (between juveniles and adults in the same treatment or between different treatments for *Daphnia* of the same age class in the experiments) of the five-step experiments, a regression analysis was performed. Mean *Daphnia* depth was used as the result of a single experiment, whereas the independent variables were: the treatment (each of the four combinations of the presence or absence of food and predation risk gradients), age class (adults or juveniles) and column (left or right). Overall population density was the covariate (Table I).

**Table I:** Analysis of regression (*P*, *F*, *df* and *error df*) between *Daphnia* mean depth in each single experiment and the overall population density used in this experiment

Comparison	Slope of the regression	Comparison of	
		Slopes	Intercepts
	*P F*_*df;Edf*_	*P F*_*df;Edf*_	*P F*_*df;Edf*_
Juveniles and adults in the absence of gradients	**0.0019 13.29**_**1;19**_	0.2212 1.62_1;16_	0.0351 5.25_1;17_
Juveniles and adults in the gradient of food alone	**0.0029 13.21**_**1;19**_	0.3237 1.04_1;16_	0.0465 4.62_1;17_
Juveniles and adults in the gradient of predation risk alone	0.3229 1.04_1;18_	0.9880 0.00_1;15_	**<0.0001 35.12**_**1;16**_
Juveniles and adults in the presence of both gradients	0.0364 5.00_1;22_	0.2172 1.63_1;19_	**<0.0029 11.54**_**1;20**_
Juveniles in the absence of gradients and in the presence of food gradient alone	0.0163 7.02_1;19_	0.8930 0.02_1;16_	0.0497 4.46_1;17_
Adults in the absence of gradients and in the presence of food gradient alone	**0.0031 13.02**_**1;19**_	0.7401 0.11_1;16_	0.2419 1.47_1;17_
Juveniles in the absence of gradients and in the presence of predation risk alone	0.2977 1.15_1;19_	0.7713 0.09_1;16_	**<0.0001 87.37**_**1;17**_
Adults in the absence of gradients and in the presence of predation risk alone	0.3243 1.03_1;18_	0.7420 0.11_1;15_	**<0.0001 98.20**_**1;16**_
Juveniles in the presence of food gradient alone and in both gradients	0.1339 2.44_1;21_	0.6781 0.18_1;18_	**<0.0001 49.98**_**1;19**_
Adults in the presence of food gradient alone and in both gradients	0.4519 0.59_1;20_	0.9518 0.00_1;17_	**<0.0001 86.20**_**1;18**_
Juveniles in the presence of predation risk alone and in both gradients	0.1293 2.50_1;21_	0.7669 0.09_1;18_	0.6293 0.24_1;19_
Adults in the presence of predation risk alone and in both gradients	**0.0035 11.28**_**1;19**_	0.6468 0.22_1;16_	0.8582 0.03_1;17_

The 12 comparisons of the distribution of *Daphnia longispina* at different population densities between juveniles and adults in the same treatment or between different treatments for *Daphnia* of the same age class in the experiments were conducted to verify the model predictions. The level of significance was fixed at *α* = 0.00417 after Bonferroni’s adjustment for 12 comparisons. Significant differences are marked in bold.

A regression analysis was performed to assess the difference in the vertical distribution of *Daphnia* in the five-step experiments and the simulations in the two versions of the model assumptions (the functional response is consistent with type II or III). Mean *Daphnia* depth was used as the result of a single experiment, treatment and the apparatus column studying the vertical distribution of the zooplankton (left or right) were the independent variables, and overall population density was the covariate (Table II). All the statistics described above were performed with Statistix 9 software.

**Table II:** Analysis of regression (*P*, *F*, *df* and *error df*) between *Daphnia* mean depth in the experiments and the overall population density for 16 comparisons between the simulations and experiments were conducted to verify the model predictions for juvenile and adult *Daphnia*, all of the combinations of the four treatments (presence or absence of the gradient of food concentration and the gradient of predation risk) and both versions of the model assumptions (type II or III functional response)

		Treatment	No gradients	Food gradient alone	Predation gradient alone	Both gradients
Age class	Assumptions	Comparison	*P F*_*df;Edf*_	*P F*_*df;Edf*_	*P F*_*df;Edf*_	*P F*_*df;Edf*_
Juveniles	type II	Slopes	0.0632 4.3_1;14_	0.6105 0.3_1;16_	**0.0070 9.6**_**1;16**_	**0.0079 3.4**_**1;20**_
		Intercepts	**<0.0001 890.4**_**1;15**_	**<0.0017 11.8**_**1;17**_		
Juveniles	type III	Slopes	0.0795 3.4_1;14_	0.6950 0.3_1;16_	0.0243 6.2_1;16_	0.3243 1.0_1;20_
		Intercepts	**<0.0001 814.5**_**1;15**_	**<0.0019 10.9**_**1;17**_	**<0.0001 127.3**_**1;17**_	0.0847 3.3_1;21_
Adults	type II	Slopes	**0.0002 22.2**_**1;18**_	0.0247 6.1_1;16_	**0.0113 5.4**_**1;14**_	**0.0097 8.4**_**1;18**_
		Intercepts		0.0969 3.1_1;17_		
Adults	type III	Slopes	**<0.0001 31.4**_**1;18**_	0.0266 6.1_1;16_	0.2397 1.51_1;14_	0.0948 3.1_1;18_
		Intercepts		0.1013 2.9_1;17_	**<0.0001 184.8**_**1;15**_	0.0465 4.5_1;19_

The level of significance was fixed at *α* = 0.0125 after Bonferroni’s adjustment for four comparisons, each within the treatments (data between treatments were not compared) separately for juveniles and adults. Significant differences are marked in bold. Statistics for the intercepts are not shown when the difference between the slopes is significant

## Results

### Experiments to determine the parameters for the model

#### The growth rate experiments

The individual growth rate (*GR*) of *Daphnia* was only affected by the concentration of algal food (i.e. *GR* was greater at higher food concentration, *a*_1_ and *a*_2_ ≠ 0 in Eq. (15), Table 1 in Appendix 3), and the interaction between age class and light intensity (i.e. light intensity, as the main component of predation risk, hampered *GR* more strongly in adults than in juveniles, *u* ≠ 0 in Eq. (15), Table 1 in Appendix 3). The intercept was also ≠0 (Eq. (15), Table 1 in Appendix 3), which seems to be an artifact as the result of adapting an inappropriate statistical model or due to the presence of bacterial food in the experimental media. The choice of the simplified variants of Eq. (15), which were introduced to the model, and the parameterization of these functions, are described in Appendix 3.

#### The experiments for assessing the relationship of RD and the intensity and spectral composition of the light

The *RD* of the fish was greater at higher light intensity (*k* ≠ 0 in Eq. (16), Table 2 in Appendix 3) and was greater for adult than for juvenile *Daphnia* (*l* ≠ 0 in Eq. (16), Table 2 in the Appendix 3). *RD* was lower in the red band of light than in the green band (*α* ≠ 0 in Eq. (16), Table 2 in the Appendix 3), was similar in the green and blue bands (*φ* = 0 in Eq. (16), Table 2 in Appendix 3), and was greater in the blue band than in the red band (*α* ≠ 0 in Eq. (17), Table 2 in Appendix 3). The choice of the simplified variants of Eq. (16) and (17), which were introduced to the model, and the parameterization of these functions are described in Appendix 3.

#### The experiments for assessing the relationship of *per capita* mortality risk from fish and *Daphnia* population density

**Fig. 1.**
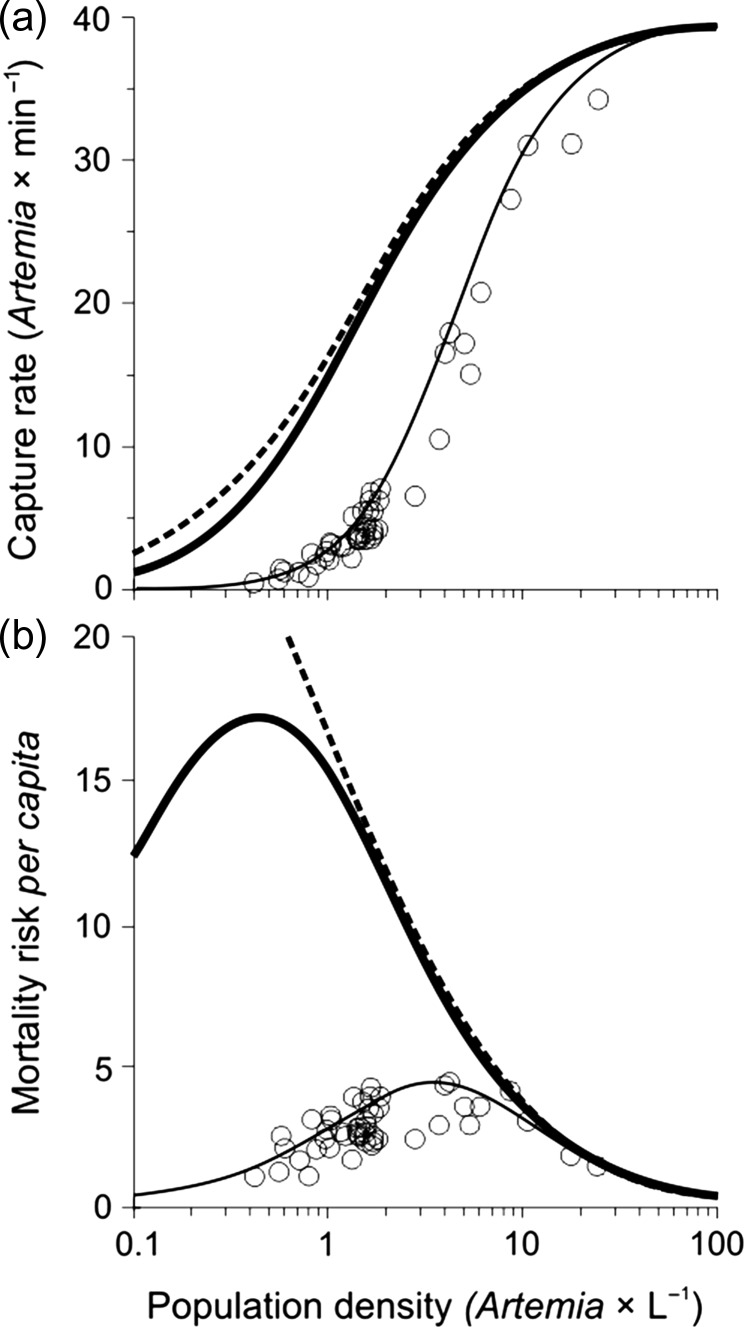
Consumption rate of rudd (**a**) and mortality risk *per capita* of its prey (*Artemia* naupli as a substitute for juvenile *Daphnia*—both of a similar body mass, (**b**)) at different densities of *Artemia* population in the experiments to determine the parameters for the model. Each point represents the mean value for one experiment (feeding session). Best fits were adjusted to the results based on equations (11–13), which introduced either type II or III functional response to the model, depending on the value of parameter *γ*, as the threshold prey density above which foraging is initiated (*γ* = 0 for type II, and *γ* > 0 for type III, Appendix 1). The thin continuous line shows the best fit for the type III response (which was used to calculate juvenile mortality risk in the model), the dashed line shows the type II response with corresponding parameters, and the thick continuous line shows the type III response used in the model for adult *Daphnia*—with the value of *γ* decreased by two orders of magnitude compared to the best fit (from 3.4158 to 0.0342) (see Appendix 3).

The type of functional response of the fish was consistent with type III rather than type II (*γ* and *n* ≠ 0 in Eq. (20), Table 3 in the Appendix 3, Fig. 1a), which was even more apparent when the data were transformed to the relationship between *per capita* mortality risk and density of zooplankton (Fig. 1b). The swimming speed of fish during foraging (averaged for the experiments with different densities of zooplankton) was 5.62 ± 1.99 cm × s^−1^.

#### The experiments for assessing the slowdown of *Daphnia* growth rate in the food gradient resulting from imperfect knowledge

The individual growth rate (*GR*) of *Daphnia* was greater in the treatment with homogeneously distributed algal food than in the treatment with the gradient of algal food (three-way ANOVA, *F*_1.44_ = 14.10, *P* = 0.0006), which suggests that *Daphnia* are mistaken in their assessment of the profitability at each depth, and that these mistakes generate costs. Although the difference was apparent for both age classes (4.7% for adults and 9.2% for juveniles), Tukey’s *post hoc* test revealed a significant difference only for juveniles. The percentage differences were used to assess the value of the parameter *æ* in Eq. (15) and (16) in Appendix 1 for both age classes (*æ*_*F,juv*_ = 0.19 for juveniles, and *æ*_*F,ad*_ = 0.08 for adults), which introduced the consequences of the fallibility of *Daphnia* in assessing food profitability on their depth selection behavior. The standard deviation of mean *GR* for individuals in the gradient of food concentration was greater than for individuals in the column with homogeneously distributed food (respectively, 0.023 and 0.013 for juveniles, and 0.023 and 0.016 for adults).

### Experiments conducted to verify the model predictions

The body length of *Daphnia* introduced to the experiments was 0.678 ± 0.057 mm for the juveniles (2.5-day-old), and 1.129 ± 0.078 mm for the adults (5.5-day-old).

**Fig. 2.**
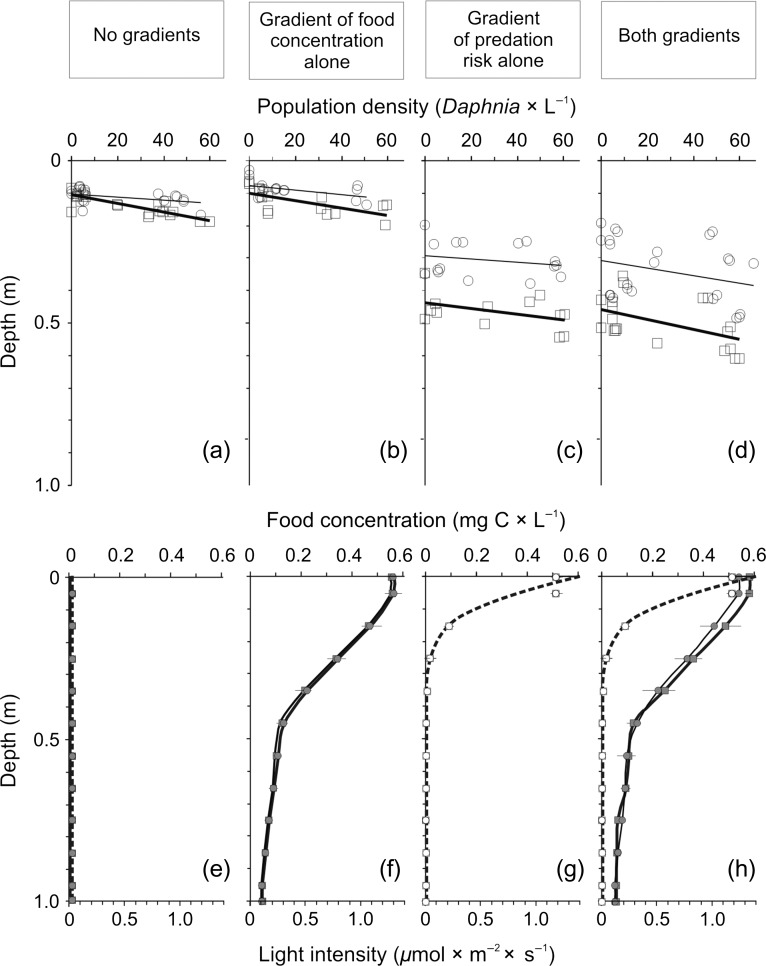
Vertical distribution of juvenile (thin lines and circles) and adult (thick lines and squares) *Daphnia* at their different population densities in each of the four treatments (**a**–**d**) and the shape of the gradients for respective treatments (mean ± SD, (**e**–**h**), continuous lines for the gradient of food concentration and dashed lines for the gradient of light intensity): in the absence of any gradients (a, e), in the presence of the food gradient alone (b, f), in the presence of the gradient of predation risk alone (c, g) and in the presence of both gradients (d, h) in the first series of experiments conducted to verify the model predictions performed in the apparatus comprised of twin columns to study the vertical distribution of zooplankton (Maszczyk, 2016). The gradient of predation risk was shown only as the gradient of light intensity, but it also consisted of two other components, i.e. the presence of kairomones and alarm substances, which was assumed to be evenly distributed throughout the columns, and the presence of the light spectrum gradient, which was the same in all the experiments performed in the apparatus and is shown in Fig. 1 of the earlier study (Maszczyk, 2016).

The distribution of *Daphnia* in the experiments was affected by the density of their populations (significant regression slope, linear regression) in 4 of the 12 cases for the plotted data: (i) for juveniles and adults in the absence of gradients, (ii) for juveniles and adults in the food gradient alone, (iii) in the absence of any gradients and in the presence of the food gradient alone for adults and (iv) in the presence of the predation risk gradient alone and in both gradients for adults (Table I, Fig. 2). In each of the four cases, *Daphnia* selected deeper strata at higher densities. While in the first three cases, this effect may be due to increased interference competition between individuals, in the fourth case, this may also have been due to greater information on predation risk in higher densities, which is consistent with the results obtained in the experiments for assessing the shape of the functional response, that is, the shape is consistent with the type III response.

The effect of density was similar for adults and juveniles in each of the four treatments and between treatments for each of the two age classes (all 12 comparisons of slopes were not significant, Table I, Fig. 2).

The difference in the mean depth selected by *Daphnia* was significant in 6 of the 12 comparisons: adult individuals resided in a deeper layer than juveniles in the gradient of food concentration alone and in both gradients; adult and juvenile individuals resided at a deeper layer in the gradient of predation risk alone than in the absence of any gradients; and, adult and juvenile individuals resided in a deeper layer in both gradients than in the gradient of food alone (Table II, comparison of intercepts, linear regression, Fig. 2).

### Simulations of virtual Daphnia distributions and the confrontation of the distribution from the experiments and simulations

**Fig. 3.**
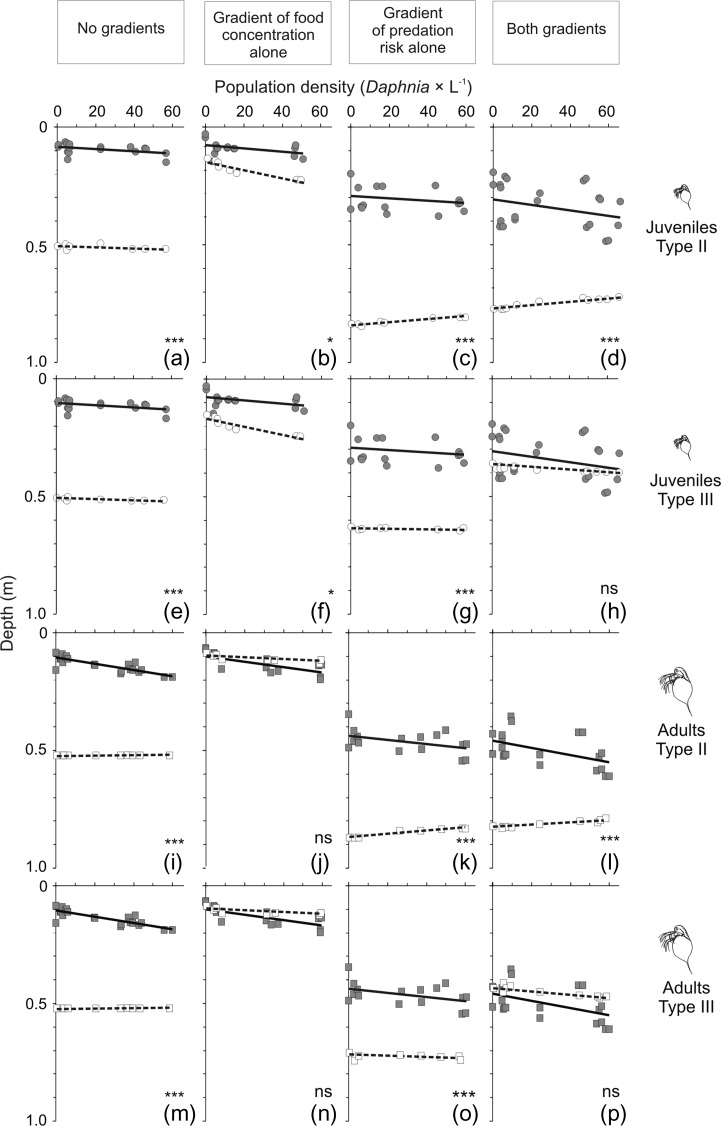
Distribution of juvenile and adult *Daphnia* at their different densities in each of the four treatments: in the absence of any gradients (**a**, **e**, **i**, **m**), in the presence of the food gradient alone (**b**, **f**, **j**, **n**), in the presence of the gradient of predation risk alone (**c**, **g**, **k**, **o**) and in the presence of both gradients (**d**, **h**, **l**, **p**) in the experiments conducted to verify the model predictions (continuous lines and filled circles, each represents the mean depth in one of the two repetitions from a single experiment) and in the model simulations (dashed lines and open circles, each represents the mean depth in a single variant of simulation) for each of the two versions of the model assumptions: (i) the functional response of fish is consistent with type II (a–d and i–l) and (ii) the functional response of fish is consistent with type III (e–h, m–p). Significant differences in the intercepts for the results of the experiments and simulations are shown as * at *P* < 0.013, *** at *P* < 0.00013, ns indicates no significance. Detailed statistics are shown in Table II.

In the absence of any gradients (Fig. 3a and e, open symbols and dotted lines), virtual *Daphnia* of both age classes distributed evenly in the water column in the whole range of population density. Therefore, the mean depth selected by individuals in each simulation was close to 0.5 m. In the food gradient alone (Fig. 3b and f, open symbols and dotted lines), *Daphnia* of both age classes selected the food rich subsurface layers, but selected slightly greater depths at higher than lower densities due to the need to avoid interfering with other individuals. In the gradient of predation risk alone (Fig. 3c and g, open symbols and dotted lines), *Daphnia* resided in deeper layers than in the other three treatments. In both gradients (Fig. 3d and h, open symbols and dotted lines), *Daphnia* resided in deeper layers than in the presence of the food gradient alone, and closer to the surface than in the presence of the gradient of predation risk alone. Comparing the depth selected in the absence of any gradients, they dwelled more deeply in the scenario assuming the type II response, and closer to the surface for the scenario assuming the type III response. In the presence of predation risk alone and in both gradients, *Daphnia* resided in deeper layers in the scenario of the model assumptions with the type II than with the type III response. Also in both treatments, adults resided slightly more deeply than juveniles, and *Daphnia* of both age classes resided in deeper layers at low population densities in the scenario assuming the type II response, and resided in deeper layers at high densities in the scenario assuming the type III response.

In the absence of any gradients (Fig. 3a, e, i, m), the mean depth selected by *Daphnia* was much greater in the simulations (open symbols and dotted lines) than in the experiments (filled symbols and continuous lines). This was apparent for both juveniles and adults (Table II). The difference in the effect of population density on the depth selected between the simulations and experiments was apparent for adults, but not for juveniles (Table II).

In the gradient of food alone (Fig. 3b, f, j, n), the difference in the mean depth selected in the simulations and in the experiments was slightly significant in juveniles, but not significant in adults (Table II). The difference in the effect of population density was not significant for any of the two scenarios of the model assumptions, either for juveniles, or for adults (Table II).

In the gradient of predation risk alone (Fig. 3c, g, k, o), *Daphnia* of both age classes resided much more deeply in the simulations generated for both scenarios of the model assumptions than in the experiments (Table II). The difference was slightly smaller for the simulations assuming the type III rather than type II response. While the difference in the effect of population density in the simulations and experiments for both age classes was significant for the model assuming the type II response, it was not significant for the model assuming the type III response (Table II).

In both gradients (Fig. 3d, h, l, p), *Daphnia* of both age classes resided in a deeper layer in the simulations assuming the type II response than in the experiments (Table II). However, the difference was not significant in the simulations assuming the type III response. While the difference in the effect of population density in the simulations and in experiments was significant for both age classes in the model assuming the type II response, it was not significant for the model assuming the type III response (Table II).

## DISCUSSION

The results of comparing the distributions obtained in the simulations and experiments supported the hypothesis of IFD with costs for *Daphnia* in overlapping gradients of food concentration and risk to visual predation. In other words, individuals (juveniles and adults) trade-off high growth rate for low mortality risk in terms of their body size and population density level. However, the distributions were similar only in both overlapping gradients for both age classes and only for adults in the food gradient alone. The greatest similarity was found for the simulations based on the scenario assuming the type III rather than type II functional response.

The hypothesis was not confirmed in the absence of any gradient because the mean depth selected by individuals of both age classes distinctly differed in the model simulations and in the experiments for the whole range of *Daphnia* population densities. The distributions in the simulation were even, which resulted from the fact that the only factor affecting the distribution of virtual individuals was the need to avoid interference competition. Although the experiments also showed an apparent effect of population density (*Daphnia* dwelled in a deeper layer and their distributions were wider at higher population densities), individuals of both age classes preferred the subsurface layers, which indicated that another factor or factors were also responsible for their depth selection behavior. The most likely explanation would be the effect of “evolutionary memory”, not considered in the model. It was motivating *Daphnia* to swim in the direction of the water’s subsurface layers. These layers are generally the most abundant in food, especially when there is a low concentration of food at the depth where an individual was located.

The hypothesis was confirmed in the presence of the food gradient alone for both age classes, that is, *Daphnia* (particularly at low population densities) selected depths close to the surface in both the simulation and the experiments.

Experiments to parameterize the model revealed another quite interesting result. In the experiments to determine error in assessing food conditions by *Daphnia*, the variability in the individual growth rate was greater for *Daphnia* searching the depths in heterogeneously rather than in homogeneously distributed algal food, which suggests that the presence of a vertical gradient of food may be a selective force for planktonic animals. Moreover, referring the obtained results from the experiments to natural conditions in a lake, the effect of error in assessing growth rate by *Daphnia* may be expected to be even greater than in the experiments, because being distributed in a miniaturized system could allow wrong decisions to be rapidly corrected. On the other hand, the effect of error could be smaller in a lake, at least when nutritional conditions (the quality and quantity of food for *Daphnia*) change unpredictably over time, that is, the subsurface layers are not always the most profitable ones (e.g. Williamson *et al.*, 1996; Lampert *et al.*, 2003).

The hypothesis was not confirmed in the presence of predation risk alone because the mean depth selected by individuals, particularly by juveniles, differed between the model simulations and the experiments for the whole range of *Daphnia* population densities. Although the effect of predation risk on depth selection was strong in the experiments, *Daphnia* mainly selected intermediate depths, while in the simulations, they selected the bottom sectors of the water column. As in the absence of any gradients, the most likely explanation for the fact that individuals of both age classes remained closer to the water surface in the experiments (than in the simulations) is the motivation of *Daphnia* to swim in the direction of the water’s subsurface layers, generally the most abundant in food, which was not considered in the model.

The difference in the depth selected in the simulations and experiments in the presence of predation risk alone was distinct in both versions of the model assumptions, but it was less apparent for the scenario assuming the type III rather than type II functional response. Moreover, another argument suggesting that *Daphnia* are adapted to avoid predation with the type III functional response comes from the comparison of the effect of predation risk on depth selection at different population densities. It was greater at higher densities than at lower ones. Although these results are not consistent with the majority of the results obtained in earlier experimental studies on the shape of the functional response of planktivorous fish, which suggest the type II rather than III response (Bence and Murdoch, 1986; Vollset and Bailey, 2011; Murray *et al.*, 2013), they are consistent with the results obtained in other studies (Townsend and Risebrow, 1982; Winkler and Orellana, 1992) and with the results obtained in this study in the experiments to parameterize the model.

Additionally, the results from the experiments for the model parameterization confirmed the results obtained in earlier studies (Utne-Palm, 1999; Downing and Litvak, 2001) that the RD of planktivorous fish (as a behavioral proxy for visual prey detection) and thus, the predation risk to zooplankton, would depend not only on light intensity but also on its spectral composition. Our results showed that the RD was smaller in the wavelength range of 656–880 nm, corresponding to red light, than in the range of shorter wavelengths of visible light, corresponding to blue and green light.

In both overlapping gradients, the hypothesis was supported because the distributions of *Daphnia* of both age classes in the experiments reflected the predictions of the model simulations assuming the type III functional response. The mean depth selected by individuals differed between the experiments and model simulations when the type II response was assumed. Moreover, in both simulations based on the model assuming the type III response and in the experiments, adult individuals selected greater depths than juveniles, particularly at higher population densities. Therefore, the results obtained in both gradients suggest that *Daphnia* takes into account both body size and population density in assessing the most profitable location.

The results of the comparison between the simulations and experiments regarding depth selection suggest that *Daphnia* rely more strongly on actual conditions in terms of predation risk than food availability. This may be so because predation risk is the stronger selection factor. This is seen in lakes with the presence of fish, where *Daphnia* population density is limited by mortality due to fish predation to a greater extent than by starvation. It is also possible that food conditions are more constant in natural conditions, as the maximum algal density is observed in the near-surface layers of most lakes. In contrast, predation risk is much more variable because it changes with the light conditions between day and night.

The literature provides numerous examples of experimental studies for a variety of animal groups showing that they assess their population density, trading-off the maximization of net energy gain and minimization of predation risk when searching for the most profitable location. However, most of these studies are only descriptive (qualitative). They revealed that in the presence of predation risk, individuals change their location, selecting a less risky and less profitable patch in terms of food, but they do not provide evidence that in the equilibrium state of distribution, each individual selects a location offering the highest possible fitness gain (e.g. Lima and Dill, 1990; Larsson and Lampert, 2012). Quantitative evidence on the trade-offs in density-dependent predation risk and density-dependent food gradients aimed at finding the best location are much less numerous (e.g. Lima *et al.*, 1985; Gilliam and Fraser, 1987). Usually, these studies consisted of a two-step procedure, in which a quantitative model (usually one of the versions of the IFD) was used only in the first step to predict the distribution of individuals in the gradient of input or concentration of food alone. In the second step, animals were additionally exposed to information (visual or chemical) on predation risk (usually stronger in the most food profitable location). Then, either the modification of the distribution was quantitatively assessed compared to the distribution in the first step (Cowlishaw, 1997; Heithaus and Dill, 2002; Wirsing *et al.*, 2007) or the energetic equivalent of the risk was assessed, that is, an additional portion of food was assessed that must be added to the risky location so that the distribution returned to the state before this information appeared (Abrahams and Dill, 1989; Grand and Dill, 1997; Brown and Kotler, 2004). Our study seems to be one of the very few examples (e.g. Skalski and Gilliam, 2002) providing quantitative evidence on the density-dependent trade-offs using an optimization model and experimental verification of the model predictions for both the food gradient alone and for overlapping gradients of food and predation risk.

## Supplementary Material

Supplementary Data

Supplementary Data

Supplementary Data
