# Supplementary material for: Ideal free distribution of Daphnia under predation risk—model predictions and experimental verification
Source: J Plankton Res. 2018 Jul 3;40(4):471–85. doi: 10.1093/plankt/fby024 (PMC6055580; doi:10.1093/plankt/fby024)
Supplement: Supplementary Data [file fby024_appendix_1_15-05-2018.doc]

**Appendix 1 (Calculating the fitness of an individual and justification of using the individual-based model)**

**I.** **Calculating the fitness of an individual**

The fitness of an individual was expressed (according to the modified equation *M_t_ Gr^-1^* proposed by Werner and Gilliam, 1984) as:

Fitness = *Gr* *M_t_ ^-1^* (1)

where: *Gr* – individual growth rate (day^-1^), and *M_t_* – total *per capita* mortality risk (day^-1^).

Although several drawbacks of the *M_t_ Gr^-1^* minimizing rule as a proxy of fitness have been identified (eg. Railsback et al. 1999), they do not apply to our study. **First**, the difficulty of applying the rule is due to the fact that the value of a fraction would tend to have very high values when *M_t_* is close to 0. It also behaves strangely when *Gr* is equal to 0 or negative. However, in our model, the fraction was relatively small, and neither *Gr*, nor *M_t_* were equal to 0 in any case. *Gr* was always positive even in the absence of algal food, which is apparent in the experiments for the model parameterization (Step 3a). *M_t_* was never close to zero even in the absence of mortality risk due to fish predation, because *M_t_* also depended on mortality risk from other sources (*M_b_*, see Eq (3) in Appendix 1). **Second**, it was pointed out that the rule could be applied only to individuals in a particular life history stage, that is, to individuals which grow and do not reproduce (Railsback et al. 1999). This was exactly the case in our study. Individuals of both age classes were before their first reproduction. Older individuals were at a stage just before depositing their first clutch of eggs in the brood pouch. **Third**, the difficulty of applying the rule in individual-based models may be due to the fact that *Gr* and *M_t_* are averaged values for all individuals in the cohort, therefore they do not take into account intra-individual variability. However, this difficulty does not apply to the IFD models, which assume that individuals (juveniles or adults) are identical (in each sector food is partitioned equally among individuals) and all have the same fitness at the state of equilibrium distribution. **Finally**, since Railsback et al. (1999) suggested that the effect of mortality on fitness is underestimated in the original formula, we modified the formula by shifting the *Gr* and *M_t_* between the nominator and denominator (in the *Gr* *M_t_ ^-1^* formula).

The relationship between individual growth rate and algal food concentration, *Daphnia* density, the presence or the absence of predation threat and age class of *Daphnia* (juveniles or adults) was expressed by Eq. (2).

*Gr* = (*a_1_* (1-*e ^a2^* *^Fconc^*)) + (*b* *D_D_*) + (*u* *L_int_*  *A*) + *f* (2)

where: *F_conc_* – food concentration (mg C × L^-1^), *D_D_* – *Daphnia* density (ind. × L^-1^), *L_int_* – light intensity (*µ*mol × m^-2^ × s^-1^) as a proxy of the information of predation threat, which affects phenotypic response of *Daphnia*, *A* – age class (*A* represents the dummy variable with 0 for juveniles and 1 for adults), *a_1_*, *a_2_*, *b*, *u*, *f* are the parameters (Table 1 in the Appendix 1).

Total *per capita* mortality risk was expressed as:

*M_t_* = *M_f_* + *M_b_* (3)

where: *M_f_ – per capita* mortality risk due to fish predation, *M_b_* – *per capita* mortality risk due to other sources (e.g. invertebrate predation).

*Per capita* mortality risk due to fish predation was expressed as:

*M_f_* = *C* *N_f_* *N_D_*^-1^ = *C D_f_* *D_D_*^-1^  (4)

where: *C* – consumption rate of one fish (ind. × min^-1^), *N_f_*  – number of foraging fish, *N_D_* = number of *Daphnia* in a single sector of the column, *D_f_* – density of foraging fish, *D_D_* – density of *Daphnia* (ind. × L^-1^).

The relationship between *C* of a single fish (and in turn, the *per capita* mortality risk in the measure of fitness) and *Daphnia* body size (at a given age) and its population density was expressed by the disk equation (Holling 1959), modified by Gliwicz and Wrzosek (2008):

(5)

where: *Y – Daphnia* density in the fish’s visual field volume, *w*(*Y*) – detectability of prey, *e_r_* – encounter rate of fish in the visual field volume as the amount of water searched by the predator, *D_D_* – *Daphnia* density, and *T_h_* – handling time (Table 1), defined as the shortest time between two captures of a prey item. It was assumed that *T_h_* does not depend on *Daphnia* density and is the same for both age classes. The encounter rate is equal to the amount of water searched by the fish in time unit, which may be seen as a cylinder with the radius equal to the reaction distance (Eggers 1977) and was expressed as:

*e_r_* = *V* π *RD*^2^  (6)

where: *V* – fish swimming speed (Table 1) measured in "The experiments for assessing the relationship of *per capita* mortality risk from fish and *Daphnia* population density", and *RD* is the reaction distance towards prey of a given age class defined as the maximal distance at which a fish can spot and attack its prey.

The relationship between *RD* and light intensity, which covers the whole spectrum in the visible range (360-880 nm), was expressed in the equation (7):

*RD* = *e^d^ e^A^* *^l^* (*e^E/k α^* *L_int,B_* + *L_int,G_* + *L_int,R_*)*^k^* (7)

where, *L_int,B_* – the intensity of the blue light, *L_int,G_* – the intensity of the green light, and *L_int,R_* – the intensity of the red light. *A* - is an age of *Daphnia* (dummy variable equal 0 for juveniles and 1 for adults), and *d*, *k*, *l*, *α* are parameters (Table 1). It was assumed that the effect of the light in one of the three ranges is the same in the presence and the absence of light in the other two ranges.

The detectability of prey *w*(*Y*) in equation (5) was expressed as:

(8)

where: *γ* – the threshold *Daphnia* density in the visual field volume, in which foraging is initiated (*γ* = 0, and *w*(*Y*) = 1 for the type II functional response, and *γ* > 0 for the type III functional response), *n* – the slope of the transition from the foraging (at low *Daphnia* density) and non-foraging (at high *Daphnia* density) phase. *Y* was expressed as:

$Y=\frac{2}{3} \pi RD_{t}^{3} D_{D}$ (9)

The probability of making errors in assessing the growth rate (different for juveniles and adults) and therefore the depth selected by *Daphnia*, was introduced to the model using the threshold difference of food concentration that can be detected by an individual. We assumed that if an individual stays in a sector with food concentration *F_0_*, then there was some other concentration *F_1_* that if the food concentration in the neighboring sector was equal to *F_1_*, then it was identified by the animal as different in 50% of the cases. The absolute difference between these two concentrations for individuals of an *A* class (juveniles or adults) was expressed as:

*æ_F, A_* = |*F_1_* - *F_0_*| (10)

We assumed that *Daphnia* is omniscient in assessing the food level (*F_0_*) in a current sector, but make errors in assessing the food level in neighboring sectors. If the real value of food concentration in the neighboring sector is *F_obs_*, the individual categorizes it into one of two classes: *F_underest_* or *F_overest_* (*F_underest_ = F_0_* + *2k æ_F, A_, F_up_ = F_0_* + *2(k + 1) æ_F, A_* (where *k* is the integer such that *F_underest_ ≤ F_obs_ ≤ F_overest_*) and chooses between them with the probability proportional to the difference between *F_obs_* and *F_down_* or *F_up_*:

*P(F_overest_|F_0_) =|F_overest_-F_obs_|/(2 æ_F, A_*) (11)

The probability of making errors in assessing the mortality risk (different for juveniles and adults) and therefore the depth selected by *Daphnia*, was introduced to the model using the assumption that the light intensity *I_0_* in the current sector is the threshold intensity *I_1_* (*I_1_ < I_0_*), which is identified by the animal as different in 50% of the cases (similar reasoning as in food concentration). To find the relationship between *I_1_* and *I_0_*, we used the formula:

*I_0_* - *I_1_* = *a_I_ I_0_* (12)

where: *a_I_* = 0.21 (Ringelberg et al. 1967). After calculating the logarithm of this equation and assuming the symmetry, we get:

|ln(*I_0_*) - ln(*I_1_*)| = *æ_I_*  (13)

for both *I_1_* > *I_0_* and *I_1_* < *I_0_*, where *æ_I_* =-ln(1 - *a_I_*).

Analogically to the food level, we assumed that *Daphnia* can precisely measure the light intensity of a current sector, and estimate the light level of a neighboring one by categorizing the light intensity there (*I_obs_*) into one of two categories: *I_underest_* and *I_overest_* (where *I_underest_ ≤ I_obs_ ≤ I_overest_* and *I_overest_* = *I*_0_ exp(2*k* *æ_I_* ) and *I_underest_* = *I*_0_ exp(2(*k*-1) *æ_I_* ). The probability of choosing *I_2k_* was assumed to be:

P(*I_2k_*|*I_0_*) = |(ln(*I_2k_*) – ln(*I_0_*)|/2 *æ_I_* (14)

**II. Justification for using the individual-based model**

At least three reasons can justify the use of the individual-based rather than the analytic model in our study. The **first** is that the effect of population density, and in turn the interactions between individuals, is one of the main topics of the submitted manuscript, which favored using the individual-based model. The justification became stronger in the revised text, since the effect of population density became the main topic of our study. The effect of interference between *Daphnia* on their distribution in the food gradient alone was weak, but apparent (see the slopes of regressions in Fig. 3). Moreover, the effect was even more apparent in the gradient of predation risk. **Second**, although analytic models are usually easier to interpret, and less of a 'black box' compared to individual-based models, in the case of our study, this reasoning seems to be weakened by the fact that we tried to avoid overcomplicating the model and simplified the rules determining the depth selection behavior of *Daphnia*. Thus the results were easy to interpret even when the individual-based model was used. The individuals in our model were identical and had only a single strategy, consisting of selecting depths offering higher fitness. This was also the reason why we could not use game theory or genetic algorithm models – they require the existence of several alternative strategies. We could introduce them by adding some fitness cost of the environment sampling, but that would be merely guesswork, as it would be extremely hard, if not impossible to parameterize this cost experimentally. **Third**, the advantage of individual-based models, compared to analytic models seems to be the greater ease with which assumptions can be weakened and more realistic ones added. In our study, it was relatively easy to add the assumption that individuals are not flawless in assessing the profitability of their environment, starting from the mechanisms of this errors, that is, from the inaccuracy of the senses. Relaxing the assumption that *Daphnia* are omniscient was also possible using the analytic model, but then a probabilistic model would have to be made, which is more difficult to analyze.

Moreover, using an analytical model would not make our article less "overwhelming", because this model would still contain all the equations that we described in our study. Finally, it could be expected that both types of approaches (individual-based and analytic) should generate similar results.

**References**

Cowgill, U. M., Keating, K. I. and Takahashi, I. T. (1985). Fecundity and longevity of *Ceriodaphnia dubia/affinis* in relation to diet at two different temperatures. *J. Crust. Biol.*, **5**, 420–429.

Eggers, D. M. (1977). The nature of prey selection by planktivorous fish. *Ecology*, **58**, 46–59.

Gliwicz, Z. M., Slon, J. and Szynkarczyk, I. (2006) Trading safety for food: evidence from gut contents in roach and bleak captured at different distances offshore from their daytime littoral refuge. *Fresh. Biol.*, **51**, 823–839.

Gliwicz, Z. M. and Wrzosek, D. (2008). Predation-mediated coexistence of large-and small-bodied *Daphnia* at different food levels. *Am. Nat.,* **172**, 358–374.

Gliwicz, Z. M., Maszczyk, P., Jabłoński, J. and Wrzosek, D. (2013) Patch exploitation by planktivorous fish and the concept of aggregation as an antipredation defence in zooplankton. *Limnol. Oceanogr.*, **58**, 1621–1639.

Holling, C. S. (1959). Some characteristics of simple types of predation and parasitism. *Canad. Entomol.*, **91**, 385–398.

Railsback, S. F., Lamberson, R. H., Harvey, B. C. and Duffy, W. E. (1999) Movement rules for individual-based models of stream fish. *Ecol. Model.*, **123**, 73–89.

Ringelberg, J., Kasteel, J. and van Servaas, H. (1967) The sensitivity of *Daphnia magna* Straus to changes in light intensity at various adaptation levels and its implication in diurnal vertical migration. *J. Comparat. Phys.,* **56**, 397–407.

Werner, E. E. and Gilliam, J. F. (1984) The ontogenetic niche and species interactions in size-structured populations*. Ann. Rev. Ecol. System.*, **15**, 393–426.

**Table 1**. Parameters used in the model with the source of data from which they were estimated, either based on the literature or on own data from the experiments for model parameterization: **A** for “The growth rate experiments”; **B** for “The experiments for assessing the relationship of reaction distance and the intensity and spectral composition of the light”, **C** for “The experiments for assessing the relationship of *per capita* mortality risk from fish and *Daphnia* population density”, and **D** for “The experiments for assessing the slowdown of *Daphnia* growth rate in the food gradient resulting from imperfect knowledge”

| Parameter | Description | Value | Unit | Source |
| --- | --- | --- | --- | --- |
|  |  |  |  |  |
| *a_1_* | Coeff. 1 for the relationship between growth rate and food conc. in Eq. 2 | 0.238 | day^-1^ | Own data from **A** |
| *a_2_* | Coeff. 2 for the relationship between growth rate and food conc. in Eq. 2 | -3.508 | L × mg C^-1^ | Own data from **A** |
| *b* | Coeff. for the relationship between growth rate and population dens. in Eq. 2 | -0.00003 | L × ind.^-1^ × day^-1^ | Own data from **A** |
| *u* | Coeff. for the relationship between growth rate and the interaction between age and light intensity in Eq. 2 | -0.025 | m^2^ × s^-1^ × day^-1^  × *μ*mol^-1^ | Own data from **A** |
| *f* | The intercept in Eq. 2 | 0.0233 | day^-1^ | Own data from **A** |
| *M_b_* | Background mortality in Eq. 3 | 9.65×10^-8^ | s^-1^ | Cowgill et al. 1985 |
| *D_f_* | Density of fish in a lake in Eq. 4 | 2.00 | ind. × m^-3^ | Gliwicz et al. 2006 |
| *V* | Swimming speed of fish in Eq. 6 | 5.62 | cm × s^-1^ | Own data from **C** |
| *d* | The intercept in Eq. 7 | -3.0789 | - | Own data from **B** |
| *k* | Coeff. for the relationship between RD and light intensity in Eq. 7 | 0.7052 | - | Own data from **B** |
| *l* | Coeff. for the relationship between RD and age in Eq. 7 | 0.2183 | - | Own data from **B** |
| *α* | Coeff. for the relationship between RD and variable *E* in Eq. 7 | -0.3999 | - | Own data from **B** |
| *T_h_* | Handling time in Eq. 5 | 0.98 | s | Gliwicz et al. 2013 |
| *γ_juv_* | Threshold density of juveniles in the visual field volume in which foraging is initiated (Eq. 8) | 3.420 | ind. | Own data from **C** |
| *γ_ad_* | Threshold density of adults in the visual field volume in which foraging is initiated (Eq. 8) | 0.034 | ind. | Gliwicz et al. 2013 |
| *n* | Slope for a transition from the foraging and non foraging phase in Eq. 8 | 0.847 | - | Own data from **C** |
| *æ_F,juv_* | Coeff. for juv. in Eq. 10 and 11 | 0.19 | - | Own data from **D** |
| *æ_F,ad_* | Coeff. for adults in Eq. 10 and 11 | 0.08 | - | Own data from **D** |
| *a_I_* | Coeff. in Eq. 13 and 14 | 0.21 | - | Ringelberg et al. 1967 |
