# Supplementary material for: Ideal free distribution of Daphnia under predation risk—model predictions and experimental verification
Source: J Plankton Res. 2018 Jul 3;40(4):471–85. doi: 10.1093/plankt/fby024 (PMC6055580; doi:10.1093/plankt/fby024)
Supplement: Supplementary Data [file fby024_appendix_2_15-05-2018.doc]

**Appendix 2 (Detailed description of the experiments to determine the parameters for the model)**

*The growth rate experiments*

The experiments were performed in a standard flow-through system comprised of 250-ml glass chambers (Stich and Lampert 1984). The chambers were placed in a water bath maintaining a constant temperature (21 ºC). A total of eight experiments were performed between March and May 2015, four experiments for each of the two age classes either in the absence or the presence of light (12:12 L:D cycle, intensity of 1.2 *μ*mol × m^-2^ × s^-1^). In each experiment, 24 chambers were used, including two repetitions for each of the 12 treatments, which were combinations of three food concentrations (0.05, 0.20 and 0.80 mg C_org_ × L^-1^) and four prey densities (8, 32, 128 and 512 ind. × L^-1^ represents respectively 2, 8, 32 and 128 ind. per chamber). The experimental media were prepared using lake water with kairomones and alarm substances and were replaced every 12 hours. Each experiment was started with 0.25- or 3.25-day-old individuals (± 0.25 day) and lasted 3 days. Before each experiment with 3.25-day-old *Daphnia*, the animals were maintained in glass containers with 2.0-*μ*m filtered lake water and fed daily with *Acutodesmus* algae at a concentration above the incipient food level (0.8 mg C_org_ × L^-1^). To determine the initial size and body mass of *Daphnia* in each experiment, 20 randomly chosen individuals were photographed under a dissecting microscope using MultiScan® measuring software, then preserved in formalin, rinsed, dried (12 h at 60 °C) and weighed on a microbalance (Orion-Cahn C-35). The same procedure was performed for all individuals at the end of each experiment. The body growth rate was determined as g = (lnC_t_ - lnC_0_) × t^-1^, where C is the body mass of an individual at the beginning (C_0_) or at the end (C_t_) of the experimental period.

*The experiments for assessing the relationship of reaction distance and the intensity and spectral composition of the light*

The integral part of the system to measure the reaction distance at a constant prey density (Bartosiewicz and Gliwicz 2011) was a narrow aquarium (66 cm height, 150 cm long and 15 cm width, V = 148.5 L) connected to a system of water pumps and tubes, allowing the rapid exchange of water in the aquarium. The system also consisted of (1) a computer-based BMS control system connected to 3 LED lamps that allowed any intensity and spectral composition of light to be set, (2) a camera connected to the computer, allowing fish foraging to be registered during the experiment, separated from the aquarium with a nontransparent curtain with small visors, and (3) a system of dispensers, which allowed new prey items to be added directly to the aquarium during the experiments. The shape of the aquarium encouraged the fish to swim parallel to the long wall of the aquarium and the plain of the camera.

A total of 36 experiments were performed between October and December 2014, including 18 experiments for each of the two age classes of *Daphnia*. Experiments for each age class included nine experiments at a lower light intensity (0.65 *μ*mol × m^-2^ × s^-1^) and nine experiments at a higher (1.20 *μ*mol × m^-2^ × s^-1^) one. For both light intensities, three repetitions were performed in three different spectra, either in the range of 400-540 nm, 541-655 nm, or 656-880 nm (representing respectively the blue, green, and red color range). Each repetition was conducted with a different pair of fish (6 fish in total). The experiments for each light spectrum for one of the two *Daphnia* age classes were carried out on different days (six experiments on six days).

Before each experimental day, the fish were acclimated to the given light intensity and spectrum for 12 h. One-half hour before each experiment, one pair of fish was placed in the system, the light intensity was adjusted and the camera was switched on. The experiments were started by placing 40 *Daphnia* in the aquarium at an initial density of 0.5 ind. × L^-1^. The density of prey during the experiments was maintained at a constant, which was achieved by having a fresh portion of ten prey individuals added as soon as ten had been captured by the two experimental fish. This was made possible by preparing portions of ten prey individuals before the onset of each new replication with a new pair of fish. Each experiment was terminated 5 min after the first capture by switching off the light in the system. Then, the camera was switched off, fish were removed, and the water with the remaining *Daphnia* was drained through a plankton net. The reaction distance, determined as the distance between the point when the fish turned towards the prey and the capture point, was measured from the archived movies using VirtualDub 1.10.4 software.

*The experiments for assessing the relationship of* per capita *mortality risk from fish and* Daphnia *population density*

The system was comprised of two sets of tanks, however, in this study only one of them was used. The tanks were connected with 12 cm diameter inter-tank windows, opened (to allow free movement of fish) or closed (to separate tanks) by rotary blinds. The setup contained 2 submerged infrared video cameras, which allowed the movements of fish inside the systems (in randomly chosen two tanks) to be monitored even under a very low light intensity. Light conditions were regulated by a computer-based BMS control system with PX 185 software connected to a system of 10 LED lamps that were allowed to automatically simulate the changes in sunlight occurring in the diurnal cycle under natural conditions. The temperature of the water in the experimental systems was controlled by water thermostats and an air-conditioning system. The system also consisted of 4 feeders (2 for each tank of the set), which allowed the addition of planktonic prey at the same time to all tanks only by two experimenters. All tanks of the system were connected to a retention tank (1000 L) and a water purification system (500 L) with a sedimentation chamber, granular biofilm, and a pump compartment in constant flow-through except during the time of experiments, when the connections were closed (Gliwicz and Maszczyk 2016). The system also had two nets held in steel rectangular frames that fit the shape of the tanks, the first for removing all fish from a tank in one haul, and the second for removing all of the remaining planktonic prey after the experiments from a tank in one haul.

All 48 experiments (feeding sessions) lasted 9 minutes, each performed on a different day with the same group of 100 fish and a new portion of 2-day old *Artemia* as a substitute for juvenile *Daphnia*. Before the experiments, the light was switched from automatic to manual mode, and the constant light intensity (1.2 *μ*mol × m^-2^ × s^-1^ 1 cm below the water surface) was assessed. The connections between the system of tanks and the water purification system were closed. Ten prey portions were prepared immediately before each feeding session by introducing the required number of prey into a 5-L calibrated container. This was achieved following enumeration of the prey in small subsamples using a dissecting microscope, in order to obtain the correct number of prey for both sections plus three 1% samples that were preserved for later detailed counts. The content of the container was divided into 10 equal portions in 10 cups, which were placed inside the feeders. Each experiment was started by switching on the cameras and by introducing portions of *Artemia* from the cups into each tank using feeders. Immediately before the end of a feeding session, a ten-step procedure was followed: (1) the visible light and cameras were switched off, (2) the connection between tanks were closed, (3) fish from one tank were transferred (to other tanks), (4) remaining prey in this tank were removed, (5) all fish were transferred from the remaining 9 tanks to this tank, (6) remaining prey in the remaining 9 tanks were removed, (7) *Artemia* samples were transferred separately from each tank into bottles, fixed with 4% formaldehyde, and stored for later enumeration under a dissecting microscope, (8) all inter-tank windows were opened to allow the fish free movement around the 10 tanks, (9) the connections between the tanks and the water purification system were opened, and (10) the light control system was set on automatic mode. After the experiments, plankton samples were counted and archived films were analysed to measure the swimming speed of fish.

The mean capture rate of a single fish was calculated as the number of prey consumed in all tanks (as the difference between the number of prey introduced into the system and the number remaining at the end of a feeding session) divided by the number of fish in the system and the duration of the experiments. *Per capita* mortality risk resulting from the activity of all fish was calculated by dividing the number of prey consumed during feeding session by the initial prey density in that session. The swimming speed of fish was measured in three experiments for 5 randomly chosen individuals in 2 tanks (3 × 5 × 2 = 30 measurements) using VirtualDub 1.10.4 software.

*The experiments for assessing the slowdown of* Daphnia *growth rate in the food gradient resulting from imperfect knowledge*

The apparatus to assess the reduction in the growth rate of *Daphnia* resulting from residing in a suboptimal location due to perceptual constraints allowed simultaneous observations to be made of changes in depth distribution with minimized constraints restricting the movements of an individual. The minimization of the constraints was achieved by: (1) orienting the columns vertically rather than horizontally, (2) by the greater volume of columns (6 L) than is found in the majority of other systems in use, (3) the lack of obstacles restricting the movement of *Daphnia* inside the columns, and (4) the presence of flow-through adjusted in such a way that it does not affect *Daphnia* depth selection (Maszczyk 2016). The setup also secured stable and reproducible vertical gradients of temperature, algal concentration, UV radiation, and predation risk, each reflecting natural field conditions, albeit in a reduced spatial scale, each independent of the other (Maszczyk 2016). Although the apparatus allowed a variety of gradients to be created, its potential was only used in the experiments to create the algal gradient with its maximum in the subsurface layers.

A total of eight experiments were performed, four experiments each for juveniles and adults. In each experiment, the same number of 18 individuals (one-day-old juveniles or four-day-old adults) was placed for three days in each of the two columns with the same constant temperature (21 ºC) and in the dark, but with different algal distributions: a gradient with the maximum food concentration in the top two sectors of one column, and in the second column, a homogeneous food distribution at a concentration corresponding to that in the top two sectors of the first column. Concentrations of algae at each depth were set using flow-through from the bottom to the top with inflows at each depth using a peristaltic pump (Ismatec® ISM 939D) pumping fresh media from 10 L containers and an outflow in the top sector. The algal concentration was monitored every six hours by taking samples from each depth and assessing the concentrations using a Turner Design TD-700 fluorometer. Experimental media were prepared using lake water with kairomones and alarm substances and were replaced every 12 hours. Before each experiment with adults, *Daphnia* were maintained in glass containers with 2.0-*μ*m filtered lake water and fed daily with *Acutodesmus* at a concentration above the incipient food level (0.8 mg C_org_ × L^-1^). The body growth rate was determined as g = (lnC_t_ - lnC_0_) × t^-1^, where C is the dry weight of an individual at the beginning (C_0_) or at the end (C_t_) of the experimental period.

**References**

Bartosiewicz, M. and Gliwicz, Z. M. (2011) Temporary intermissions in capturing prey (*Daphnia*) by planktivorous fish (*Rutilus rutilus*): Are they due to scramble competition or the need for antipredation vigilance? *Hydrobiologia*, **668**, 125–136.

Gliwicz, Z. M. and Maszczyk, P. (2016) Heterogeneity in prey distribution allows for higher food intake in planktivorous fish, particularly when hot. *Oecologia,* **180**, 383–399.

Maszczyk, P. (2016) Miniature plankton columns used to study the depth distribution of zooplankton in gradients of food, predation risk, temperature, and UV radiation. *Limnol. Oceanogr. Met.*, **14**, 210–223.
