# Supplementary material for: Ideal free distribution of Daphnia under predation risk—model predictions and experimental verification
Source: J Plankton Res. 2018 Jul 3;40(4):471–85. doi: 10.1093/plankt/fby024 (PMC6055580; doi:10.1093/plankt/fby024)
Supplement: Supplementary Data [file fby024_appendix_3_15-05-2018.doc]

**Appendix 3 (The model parameterization)**

The relationship between individual growth rate and algal food concentration, *Daphnia* density, the presence or the absence of predation threat and age class of *Daphnia* was expressed by the equation:

*Gr* = (*a_1_* (1-*e^Fconc^* *^a2^*)) + (*b* *D_D_*) + (*c* *L_int_*) + (*f* – (2 *a_1_*)) + (*o* (1-*e^Fconc^* *^a2^* ) *A*) + (*a_1_* (1-*e^Fconc^* *^r^**^A^*))

+ (*s D_D_* *A*) + (*u* *L_int_* *A*) + (*o* *A*) (15)

where: *Gr –* the growth rate (day^-1^), *F_conc_* – food concentration (mg C × L^-1^), *D_D_* – density (ind. × L^-1^), *L_int_* – light intensity (*µ*mol × m^-2^ × s^-1^) as a proxy of the information of predation threat, which affects phenotypic response of *Daphnia*, *A* – age class (*A* represents the dummy variable with 0 for juveniles and 1 for adults), *a_1_*, *a_2_*, *b*, *c*, *f*, *r*, *s*, *u*, *o* are the parameters. Additionally, two parameters (– 2 × *a_1_* and – *o* × A) were introduced for the equalization of the three exponential functions (*a_1_* × *e^Fconc^* × *^a2^*, *o* × *e^Fconc^* × *^a2^* × *A* and *a_1’_* × *e^Fconc^* × *^r^* × *^A^*).

To avoid a strong reduction of the power of the test, only interactions of age with other factors were introduced. The function was fitted to the results from “The growth rate experiments” using the nonlinear least square method. The effect of each variable and interaction was checked using Student’s T-test performed in the R v 3.2.2 statistical environment (R Core Team 2015, Table 1 in Appendix 1).

Although Eq. 15 was used to perform the statistics, to avoid overfitting, it was simplified using the Akaike criterion (AIC, Akaike 1974) before it was introduced to the model for the extrapolation and interpolation of the growth rate at different conditions.

All 262 combinations of the simplified equation, including equations with skipped one, two, *etc*. variables and interactions, as well as equations with a linear relationship between growth rate and food concentration, were fitted to the results from “The growth rate experiments” using either nonlinear (for the exponential options of the relationship between the growth rate and food concentration) or linear (for the linear options of the relationship) least square method (performed in the R v 3.2.2 statistical environment). Among all combinations, one equation was chosen (Eq. (2) in Appendix 1) with the lowest value of the Akaike criterion (-571), and the highest value of the Akaike weight (0.084).

*RD* (in equation 6 in Appendix: the model part) was expressed using the equation from Aksnes and Giske (1993), which was modified by adding the effect of the light spectrum on *RD* to the original formula (Eq. (16)):

*RD* = *e^d^ L_int_^k^ e^A^* *^l^* *e^E^**^α^* *e^B^* *^φ^* (16)

where: *L_int_* – light intensity (*µ*mol × m^-2^ × s^-1^), *A* – age class of *Daphnia* (2.5-day-old juveniles and 5.5-day-old adults), *E* and *B –* represent dummy variables corresponding to different ranges of the light spectrum (for 360-530 nm – blue light range: *E* = 0, *B* = 1, for 530-660 nm – green light range: *E* = 0, *B* = 0, and for i 660-880 nm – red light range: *E* = 1, *B* = 0), and *d*, *k*, *l*, *α, φ* are the parameters. Eq. (16) allowed us to check the difference in the effect of the green and red, as well as the green and blue light range on *RD*. To compare the effect of the blue and red light range on *RD*, an additional variant of the model was developed:

*RD* = *e^d^ L_int_^k^* *A^l^* *e^E^* *^y^* *e^G^**^φ^* (17)

where: *G* – represents the dummy variable with 1 for the green light and 0 for any other color. Both functions (Eq. (16) and (17)) were log transformed and fitted to the results from “The experiments for assessing the relationship of reaction distance and the intensity and spectral composition of the light” using the linear mixed model. The model was created using the *lme4* library in the R v 3.2.2 statistical environment (Bates et al. 2015). The effect of each variable on *RD* was checked using the likelihood ratio test with the chi^2^ distribution (Table 2 in Appendix 1).

Although Eq. (16) and (17) were used to perform the statistics, to avoid overfitting, Eq. (16) was simplified using the AIC (Akaike 1974) before it was introduced to the model for the extrapolation and interpolation of the *RD* at different conditions, to assess its effect on fitness and in turn on the depth selection of virtual *Daphnia*. All 15 combinations of the simplified equation, including equations with skipped one, two variables, *etc.*, were fitted to the results from “The experiments for assessing the relationship of reaction distance and the intensity and spectral composition of the light” using the linear mixed model. Among all combinations, one was chosen (Eq. (18)) with the lowest value of the Akaike criterion (-19.2), and the highest value of the Akaike weight (0.659):

*RD* = *e^d^ L_int_^k^* *e^A l^* *e^E^* *^α^* (18)

To determine the relationship between the consumption rate (*C*), and in turn, the *per capita* mortality risk at different densities of *Daphnia*, Eq. 5 in Appendix 1 (or Eq. 20 in Appendix 3) was fitted to the results from “The experiments for assessing the relationship of *per capita* mortality risk from fish and *Daphnia* population density”. However, due to the fact that these experiments were performed with live fish foraging on planktonic prey that were not being supplemented during the experiment, the decrease of prey density needed to be taken into account for the correct calculation of the predicted values by assessing the instantaneous prey number (density) and instantaneous consumption rate. First, the rate of the decrease of prey numbers (density) in each experiment was expressed as:

$\frac{dN_{D}}{dt}=-N_{f} f\left(N_{D}\right)$ (19)

where: *N_f_* – the number of fish in the experiment, *f*(*N_D_*) – functional response (i.e. the relationship between the number of prey eaten by a single fish per unit of time and the number of planktonic prey) under a constant prey number. Eq. (19) can also be expressed as a function of density: *D_D_* = *N_D_* × *Vol*^-1^, where *Vol* is the volume of the experimental system (*Vol* = 2000L). Second, the rate of change in prey density was expressed by the integration of Eq. 9 from Appendix 1:

(20)

where: *t_z_* – duration of *z*-experiment (i.e. feeding session, 2-15 min), *N_0D,z_* – the initial number of a planktonic prey in *z*-experiment, *N_D,z_* – the final number of planktonic prey in *z*-experiment, *T_h_* – handling time (Table 1 in Appendix: the model part), *g* and *j –* clusters of other parameters: *g* = *π* × *V* × *RD_w_^2^* × *Vol*^-1^ and *j* = 2 × *π* × *RD_w_*^3^ × (3 × *Vol*)^-1^, where *V* – swimming speed of fish (Table 1 in Appendix 1), *RD_w_* – reaction distance to *w*-age class (Table 1 in Appendix 1), and *Vol* – the volume of the experimental setup (Table 1 in Appendix 1).

Assuming the type II functional response (i.e. when *w*(*Y*) = 1), Eq. (20) is simplified to Eq. (3) from Appendix 1, therefore, the value of all parameters are known and predicted values can be calculated. To determine the relationship between consumption rate (*C*, and in turn, the *per capita* mortality risk) at different densities of *Daphnia* in the case of type III functional response, Eq. (20) (with unknown values of *γ* and *n*) was fitted to the results from “The experiments for assessing the relationship of *per capita* mortality risk from fish and *Daphnia* population density” using the nonlinear least square method. Due to the fact that it is hard to arithmetically transform Eq. (20) to a form enabling the direct enumeration of *N_D,z_* (*N_D,z_* = F(*t_z_*, *N_0D,z_*)), expected values of *N_D,z_* were assessed numerically in the statistical model using the Newton method. The effect of both parameters (*γ* and *n*) was tested using Student’s T-test (Table 3 in Appendix 3). This test was also used to determine whether the results from the experiments are closer to type II or III functional response, assuming that *γ* does not differ significantly from 0 only in the case of type II.

Due to the fact that in “The experiments for assessing the relationship of *per capita* mortality risk from fish and *Daphnia* population density” 2-day-old *Artemia* was used as the only type of prey as a substitute for juvenile *Daphnia*, parameters *n* and *γ* were assessed directly from the data of these experiments for only juvenile individuals. For adults, the same value of *n* was assumed, but the value of *γ* assessed for juveniles was divided by 100. This was done according to Gliwicz and Wrzosek (2008), who predicted that the threshold density of juvenile and adult *Daphnia* below which fish stop foraging can even differ by two orders of magnitude.

The value of *æ_F, A_* (Eq. (10) in Appendix 1) was estimated based on the results from experiments on “The slowdown of *Daphnia* growth rate in the food gradient resulting from imperfect knowledge”. The growth rate of *Daphnia* (juvenile or adult) at selected depths in the treatment with the food gradient was compared to the growth rate of individuals (of the same age class) distributed in the treatment with a homogenous food concentration (equal to the concentration in the top sector of the treatment with the gradient – the mean carbon concentration was 0.60 mg C × L^-1^ for juveniles and 0.59 mg C × L^-1^ for adults). The treatment with homogenous food simulates the scenario of “flawless” *Daphnia*, because the growth rate of individuals in this treatment was highest irrespective of the depth they selected. The treatment with the gradient simulates the scenario of “mistaken” *Daphnia*, because the growth rate depends on the potential errors of individuals in assessing the growth rate in different sectors. The results of these experiments were used to calculate the difference in the growth rate between “flawless” and “mistaken” *Daphnia*:

*Diff* = 100 % (*GR_homo, A_* – *GR_hetero, A_*) *GR_homo, A_*^-1^ (21)

where *GR_homo, A_* – growth rate of *Daphnia* of age class *A* selecting depths in the treatment with the homogenously distributed food concentration, and *GR_hetero, A_* – growth rate of *Daphnia* of age class *A* selecting depths in the treatment with the gradient of food concentration. The difference was 9.2% for juveniles and 4.7% for adults. To estimate the real value of parameter *æ_F, A_* (Table 1 in Appendix 1), a series of simulations was conducted separately for each of the two age classes. The conditions of the simulations were set to the conditions of the experiments (the same food concentrations, age classes, population densities). Each simulation lasted for 200 time steps, but to obtain stable *Daphnia* distributions, only data for the last 100 time steps were used to calculate the mean growth rate. The difference between growth rates (Eq. (21)) was then calculated for the data obtained in the simulations, and was compared to the data from the experiments. The linear regression of the difference in growth rates against the value of *æ_F, A_* used in the simulations was calculated and the point where this line crosses the difference obtained from the experiments was used to estimate the real value of *æ_F, A_* (Table 1 in Appendix 1). To assess the difference in the growth rate of *Daphnia* (juveniles and adults) in the treatments with homogeneously and heterogeneously distributed algal food in “The experiments for assessing the slowdown of *Daphnia* growth rate in the food gradient resulting from imperfect knowledge”, the three-way ANOVA with Tukey's *post hoc* test was performed with the treatment (homogeneously or heterogeneously distributed algal food), age class (juveniles or adults) and column (left or right) as independent variables.

**References**

Akaike, H. (1974) A New Look at Statistical Model Identification. *IEEE Trans. Aautomat. Contr.,* **19**, 716–723.

Aksnes, D. L. and Giske, J. (1993). A theoretical model of aquatic visual feeding. *Ecol. Model.*, **67**, 233–250.

Bates, D., Maechler, M., Boler, B. and Walker, S. (2015). Lme4: Linear mixed-effects models using Eigen and S4. *R package version*. **1**, 1–9.

Gliwicz, Z. M. and Wrzosek, D. (2008). Predation-mediated coexistence of large-and small-bodied *Daphnia* at different food levels. *Am. Nat.*, **172**, 358–374.

R Core Team. 2015. R: a language and environment for statistical computing. R Foundation for Statistical Computing, Vienna, Austria. https://www.r-project.org

**Table 1.** The results of Student's T-test for the best fit of the results of “The growth rate experiments for model parameterization” to Eq. (15). The level of significance was fixed at *α* = 0.005 after Bonferroni’s correction for 10 tests (*α* = 0.05/10). Significant differences are presented in bold

| Parameter | Value | Standard error | Value of Student’s  *t*-test | *P* |
| --- | --- | --- | --- | --- |
|  |  |  |  |  |
| *a_1_* | 0.2408 | 0.0162 | -14.859 | **< 0.0001** |
| *a_2_* | -2.7720 | 0.7670 | -3.614 | **0.0004** |
| *b* | -0.0000320 | 0.0000203 | -1.578 | 0.1166 |
| *c* | -0.0107 | 0.0073 | 1.464 | 0.1452 |
| *f* | 0.2189 | 0.0141 | 15.586 | **< 0.0001** |
| *o* | -0.1308 | 0.1128 | -1.159 | 0.2481 |
| *r* | -0.4416 | 0.2936 | 1.504 | 0.1347 |
| *s* | 0.0000070 | 0.0000294 | 0.238 | 0.8120 |
| *u* | -0.0360 | 0.0105 | -3.443 | **0.0007** |
|  |  |  |  |  |

The number of degrees of freedom = 149

**Table 2.** The results of the linear mixed model for the best fit of the results of “The experiments for assessing the relationship of reaction distance and the intensity and spectral composition of the light” to Eq. (16) and (17). The level of significance was fixed at *α* = 0.0083 after Bonferroni’s correction for 6 tests (*α* = 0.05/6). Significant differences are presented in bold

| Parameter | Eq. | Parameter value | Standard error | Chi^2^ test | *DF* | *P* |
| --- | --- | --- | --- | --- | --- | --- |
|  |  |  |  |  |  |  |
| *d* | 14 | -3.0905 | 0.0391 | **-** * | **-** * | **-** * |
| *k* | 14 | 0.7026 | 0.0556 | 13.840 | 1 | **0.0002** |
| *l* | 14 | 0.2101 | 0.0461 | 6.4430 | 1 | **0.0061** |
| *α* | 14 | -0.3919 | 0.0411 | 12.465 | 1 | **0.0004** |
| *φ* | 14 | 0.0214 | 0.0514 | 0.1701 | 1 | 0.6800 |
| *y* | 15 | -0.4133 | 0.0485 | 10.324 | 1 | **0.0013** |
|  |  |  |  |  |  |  |

* Statistical analysis did not reveal any result for *d’* (intercept) = 0, which suggested a strong effect of this parameter on *RD*.

**Table 3.** The results of Student’s T-test for the best fit of the results of “The experiments for assessing the relationship of *per capita* mortality risk from fish and *Daphnia* population density” to Eq. (20) using the nonlinear least square method. Significant differences are presented in bold

| Parameter | Value | SE | Value of Student’s *t*-test | *P* |
| --- | --- | --- | --- | --- |
|  |  |  |  |  |
| *γ_juv_* | 3.4158 | 1.0278 | 3.323 | **0.00181** |
| *n* | 0.8465 | 0.1426 | 5.938 | **< 0.00001** |
|  |  |  |  |  |
